# Affinity capillary electrophoresis – mass spectrometry permits direct binding assessment of IgG and FcγRIIa in a glycoform-resolved manner

**DOI:** 10.3389/fimmu.2022.980291

**Published:** 2022-09-08

**Authors:** Christoph Gstöttner, Alexander Knaupp, Gestur Vidarsson, Dietmar Reusch, Tilman Schlothauer, Manfred Wuhrer, Elena Domínguez-Vega

**Affiliations:** ^1^ Leiden University Medical Center, Center for Proteomics and Metabolomics, Leiden, Netherlands; ^2^ Pharma Research and Early Development, Roche Innovation Center Munich, Munich, Germany; ^3^ Department of Experimental Immunohematology, Sanquin Research and Landsteiner Laboratory, Amsterdam UMC, University of Amsterdam, Amsterdam, Netherlands; ^4^ Pharma Technical Development Penzberg, Roche Diagnostics GmbH, Penzberg, Germany

**Keywords:** affinity capillary electrophoresis (ACE), mass spectrometry, FcγRIIa receptor, monoclonal antibody, glycosylation, interaction

## Abstract

The impact of antibody glycoforms on FcγRIIa activation and immune responses is poorly understood. Yet, glycoform binding assessment remains one of the major analytical challenges requiring long enrichment or glycoengineering steps. Here, we developed and applied an affinity capillary electrophoresis-mass spectrometry approach to selectively assess the binding of different antibody glycoforms to the FcγIIa receptor without the need of glycoengineering. The approach required only low microgram amounts of antibody and receptor and enables assessing the binding of high and low-abundance glycoforms. The approach indicated clear differences in binging between doubly-, hemi-glycosylated and non-glycosylated antibodies as well as for mutated (Leu234Ala, Leu235Ala – Pro329-Gly (LALA-PG)) IgG1 antibodies silenced for Fcγ binding. The LALA-PG mutated antibody showed no binding to the FcγIIa receptor (excluding potential non-specific binding effects) while the non-glycosylated IgG1 showed a strongly reduced, but still minor binding. The highest binding affinity was for the antibody carrying two complex-type glycans. Man5 glycans resulted in decreased binding compared to complex-type glycans, with the lowest binding for the IgG containing two Man5. For complex-type glycans, galactosylation showed a subtle increase in binding to the FcγIIa receptor, and sialylation showed an increase in binding for lower sialylated species. Fucosylation did not influence binding to the FcγIIa receptor. Finally, the assay was evaluated for the two variants of the FcγRIIa receptor (allotypes H131 and R131) showing highly comparable glycoform selectivity. Overall, the proposed approach allows the direct comparison of binding affinities of different antibody species in mixtures promising a fast establishment of their structure-function relationships.

## Introduction

Receptors for human IgG (FcγR) are transmembrane glycoproteins consisting of three families designated I to III. Each family consists of several genes, each encoding for a separate protein with most of them inducing an activating response *via* IgG immune complexes. Of these receptors, FcγRIIa has the widest expression, as it is present on all myeloid cell types, such as neutrophils, basophils, mast cells, eosinophils, monocytes, macrophages, dendritic cells but also platelets (1–3). Antibody binding and signaling *via* FcγRIIa receptor can induce immunological responses such as maturation of dendritic cells, cytokine and chemokine release leading further to T cell activation or platelet aggregation (2, 4). FcγRIIa contributes to the removal of immune complexes by macrophages *via* antibody-dependent cell-mediated phagocytosis (ADCP) (4). These aspects are also very important for monoclonal antibody therapies (5). In the population two different allotypes of the FcγRIIa gene can be observed, namely FcγRIIa H131 and FcγRIIa R131 (6, 7) with allotype expression influencing the clinical outcome of immunotherapy (8). The extracellular domain of FcγRIIa binds IgGs in their Fc region at the second domain of the heavy chain (CH2). Next to this binding site in the CH2 domain, the CH3 domain has been reported to be additionally involved in FcγRIIa binding (9), whereas another study on IgG1 indicated that the solely the CH2 domain interacted with FcγRIIa (10). In particular two amino acids in the lower hinge region in position L234, L235 (11, 12) and P329 in the upper hinge region appear to be critical for the interaction, and their mutation leads to a nearly complete loss of affinity (13, 14). This is especially of interest for some antibody therapeutic applications where no activation of the immune system *via* Fc receptor functions is wanted (14).

Antibodies contain a conserved N-glycosylation site in the Fc domain, close to the FcγRIIa binding interface. The influence of the Fc glycan on binding has been widely discussed. Complete deglycosylation of IgG1 monoclonal antibodies (mAbs) has been demonstrated to lead to severe decrease of affinity due to a decrease in antibody conformational stability (15–17). To study the effect of different IgG1 glycoforms, glycoengineered IgG variants (i.e. with higher populations of certain glycan moieties) have been explored, yet with controversial results. In a recent publication employing hypergalactosylated variants no difference in FcγRIIa binding compared to the reference material was observed (18), while Subedi et al. using more enriched glycoforms reported an approximately 1.5 times higher affinity for antibodies containing high amounts of galactosylation (19). A similar effect of an increased binding of galactosylated variants was found by Thomann et al. (20). Notably, results on the effect of sialylation were again divergent: Whereas Subedi et al. hardly observed any increase on affinity for sialylated glycans (19), the study of Thomann et al. found that increased sialylation resulted in increased binding to the FcγRIIa (20). Functionally, enhanced galactosylated IgG also seem to elevate platelet activation upon encounter of anti-SARS-CoV-2 IgG1 complexed with S proteins (21). These differences between studies most probably arise by the fact that glycoengineering still provides a mixture of glycoforms and that different glycoengineering strategies can lead to different glycoform mixtures (22). Up to now techniques to study the interaction between FcγRIIa (e.g. ELISA or SPR assays) cannot distinguish between co-existing glycoforms making the assessment of glycoform-specific binding challenging (18, 23). A recent alternative approach used an in-house produced FcγRIIa column combined with UV detection but still required samples enriched in specific glycoforms (20). Therefore, a glycoform-resolved, multiplexed approach would be of tremendous benefit to answer these questions. Furthermore, the influence of other common glycoforms in IgG1 therapeutics, such as high mannose structures has hitherto not been addressed and would benefit from such strategy facilitating direct analysis without the need of specific glycoforms.

**Figure 1:**
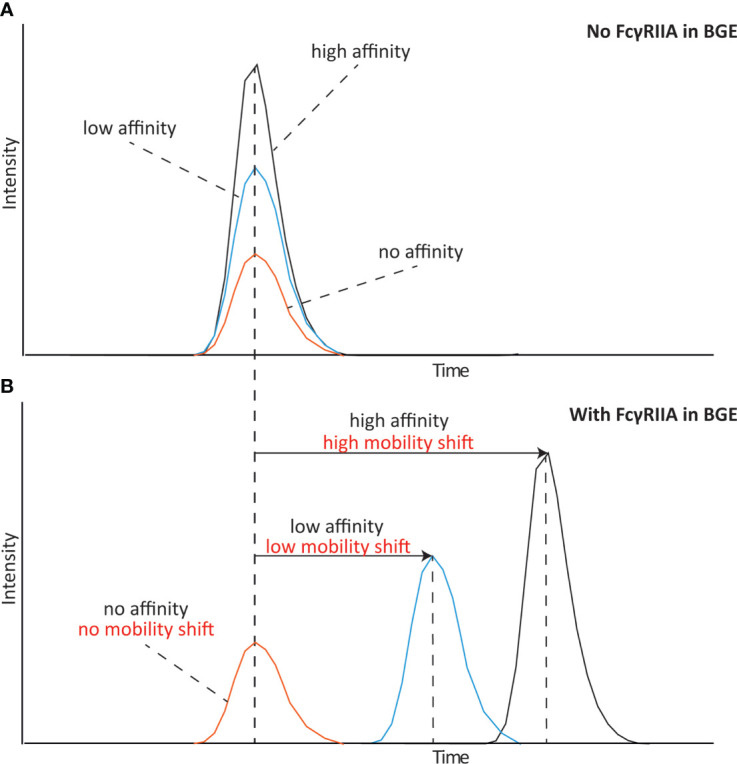
Schematical representation of an mobility shift affinity CE experiment analyzing species with high, low and no affinity to the receptor. **(A)** Affinity CE analysis without any FcγRIIa in the background electrolyte (BGE) and **(B)** analysis with FcγRIIa in the BGE.

We have recently developed a novel approach based on affinity capillary electrophoresis – mass spectrometry (CE-MS) that allows monitoring the specific binding of antibody proteoform mixtures (including glycosylated variants) to the FcRn receptor (24). Mobility shift affinity capillary electrophoresis is an approach able to determine binding affinities of specific proteoforms by monitoring the change of their electrophoretic mobility after addition of the interacting partner to the background electrolyte (25). The complex formed between the antibody and receptor presents a different electrophoretic mobility than the free species indicating binding. The schematic shown in **Figure 1** describes the behavior of species with different affinities to the Fc receptor. Whereas a species without affinity shows no change on the mobility shift, a species with high affinity to the Fc receptor shows a large mobility shift. Another species with a slightly lower affinity will also have a slightly lower mobility shift compared to the high affinity species. This way affinity CE allows to define the affinity of different species in a mixture due to their change in electrophoretic mobility. Coupling to mass spectrometric detection permits the multiplexed, parallel analysis and structural assessment of the specific species responsible of the binding. This approach opens new possibilities as multiple species (e.g glycoforms) can be monitored simultaneously. A mayor benefit of this platform is the ability to determine also medium and low affinity interactions as often observed for Fc receptors and antibodies (high nM-µM range) which can be challenging for SPR approaches.

Here, we explore the approach to study the binding affinity between FcγRIIa and different IgG1 glycoforms. Mass spectrometric detection allowed the parallel, integrated analysis of different glycoforms without the need of tedious glycoengineering for separately producing different glycoforms or the IgG molecules. IgGs with both complex-type and high mannose-type glycosylation were explored. Furthermore, we studied the impact of different glycan core structure units in respect to their affinity to the FcγRIIa, which allowed us to see an increase in the affinity with increasing size of the N-glycan core structure.

## Materials and methods

### Samples and chemicals

mAb A, C and E were provided by the Sanquin Research and Landsteiner Laboratory (Amsterdam, The Netherlands). mAb B and D as well as the FcγRIIa construct were kindly provided by Roche Diagnostics (Penzberg, Germany). To enrich in specific glycoforms, mAb C was incubated with of β(1–4)-galactosyltransferase and α2,3-sialyltransferase to obtain high level of sialyation. mAb E (low fucose) was expressed in the presence of 2-deoxy-2-fluoro-l-fucose (2FF), wherease mAb-E (high galactose) was expressed together with B4GALT1 encoding β-1,4-galactosyltransferase 1 (B4GALT1) and 5 mM d-galactose to obtain high levels of galactose. For mAb-E (high galactose + low fucose) both previous approaches were combined. The FcγRIIa consisted of the extracellular domain, linked to an AviTag and an IgG1 Fc domain, containing a LALA-PG mutation. Lysozyme from chicken egg, glacial acetic acid, 7.5 M ammonium acetate (AmAc) solution, and hydrogen chloride were purchased from Sigma-Aldrich (Steinheim, Germany). Water (ULC/MS-CC/SFC grade) was obtained from Biosolve Chimie SARL (Dieuze, France). 10 kDa Vivaspin MWCO filters for buffer exchange were purchased from Satorius (Göttingen, Germany). All mAb samples were buffer exchanged to 50 mM AmAc pH 6.8 and adjusted to a final concentration of 1 μg/μL. The FcγRIIa was also exchanged to 50 mM AmAc pH 6.8 and added to the BGE (background electrolyte) in a concentration of 2.7 μM. Lysozyme was buffer exchanged to 50 mM AmAc pH 6.8 and adjusted to a final concentration of 2 μg/μL.

### Mobility shift affinity CE-MS

Measurements were performed on a CESI 8000 instrument (Sciex, Framingham, MA) using OptiMS neutrally coated capillaries (Sciex) containing a porous tip. A neutrally coated capillary was employed to avoid protein adsorption to the capillary wall at the used conditions. The length of the capillaries was 91 cm (30 μm i.d.; 150 μm o.d). Before the first usage the capillary was flushed for 5 min (100 psi, forward) with 0.1 M HCl, followed by 10 min (100 psi, forward) with 50 mM ammonium acetate pH 3.0 and 30 min (100 psi, forward) with water. Afterwards the capillary was allowed to rehydrate for 16 to 18 h by flushing with 10 psi (forward) with water. Before each analysis the capillary was flushed for 2 min with 0.1 HCl (100 psi, forward pressure), 2 min with water (100 psi, forward), 2 min 50 mM AmAc (100 psi, forward) and 2 min (100 psi, reverse). As a background electrolyte 50 mM AmAc (pH 6.8) was used.For the ACE experiments the capillary was subsequently filled for 2 min (100 psi, forward) with the FcγRIIa receptor at the specified concentration. After that a plug of lysozyme was injected (1.5 psi, 15 s), which functions as a marker protein. Hereafter, the sample was injected (2.5 psi, 15 s) followed by a plug of BGE with or without FcγRIIa (1 psi forward, 25 s). For the separation, a voltage of 20 kV (normal polarity) with 2 psi forward pressure and 25°C was applied. The 2 psi forward pressure was applied to obtain an stable electrospray with the neutrally coated capillaries. After completion of the separation the voltage was ramped down in 5 min to 1 kV. A RSD value of 0.9% for the migration time was assessed during four consecutive injections of mAb-D.

### Mass spectrometric detection

The porous tip of the CE capillary was connected to a solariX 15 T FT-ICR-MS equipped with a ParaCell (Bruker Daltonics, Bremen, Germany) *via* a nano-electrospray ionization source. The FT-ICR-MS was operated in positive mode and a *m/z* range between 398.5 and 20000 was monitored. The dry gas flowrate was set to 1.3 L/min with a temperature of 150°C. The InSource collision energy was set to 40 V, the skimmer 1 to 180 V, skimmer 2 to 5 V and funnel 1 and 2 to 190 V and 6 V, respectively. The trapping potential was set to 2.8 V and the ParaCell DC biases ranged between 1.9 and 2.1 V. The time of flight to the detector was set to 3 ms and the ion accumulation time to 0.3 s. Each mass spectrum of the serial mode acquisition was a result of summation of 20 spectra. Data analysis was performed by using the DataAnalysis software from Bruker Daltonics (Bremen, Germany). To obtain deconvoluted average masses the maximum entropy algorithm was applied, followed by one cycle of gaussian smoothing.

## Results

### Mobility shift affinity CE-MS for monitoring the influence of antibody glycosylation on FcγRIIa binding

**Figure 2:**
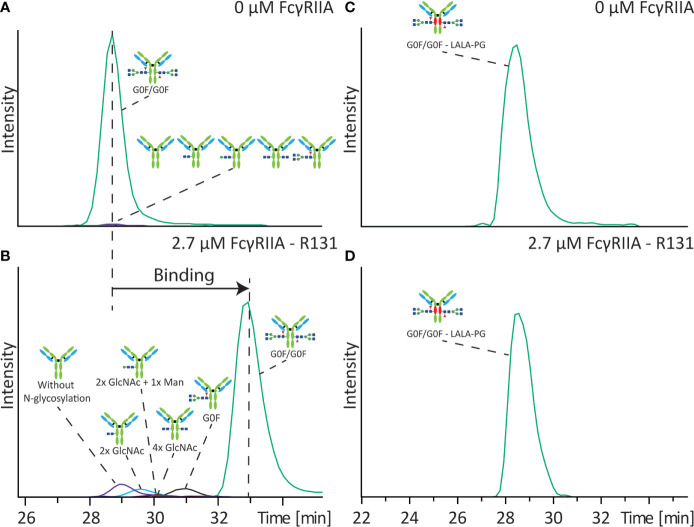
Influence of Fc N-glycan occupancy on binding towards FcγRIIa. Affinity CE-MS analysis of mAb-A **(A)** without FcγRIIa or **(B)** with 2.7 µM FcγRIIa R131 variant in the background electrolyte. In different colors are the extracted ion electropherograms (EIEs) of the illustrated antibody molecules with different degree of glycosylation. Analysis of mAb-B containing a LALA-PG mutation with **(C)** 0 µM FcγRIIa or **(D)** with 2.7 µM FcγRIIa R131 variant.

FcγRIIa and IgG exhibit a medium-low affinity interaction with reported K_D_ values between 400 and 1300 nM (18, 19), which makes it perfectly suitable to be assessed by affinity CE. The interaction of five antibodies (mAb-A to mAb-E) was studied by using a BGE of 50 mM ammonium acetate pH 6.8. To assure maximum affinity shift we evaluated various receptor concentrations above the K_D_, with 2.7 µM providing the best result with only a minor reduction of signal intensity (2-3 times) (**Figure S1**). Glycosylation has been shown to influence the interaction with the FcγRIIa (18). As first experiment we studied the binding of an antibody sample with different levels of glycosylation to the FcγRIIa R131 variant. The used sample (mAb-A) varied in N-glycosylation site occupancy (two, one or no Fc N-glycans). Analyzing mAb-A with and without FcγRIIa in the BGE allowed to determine the mobility shift of each mAb-A variant and thereby their relative binding to FcγRIIa (**Figures 2A, B** **)**. To correct for any alteration in the separation media (e.g. ionic strength, viscosity) a non-interacting protein (lysozyme) was employed as electrophoretic marker (**Figure S2**). The antibody with two complex type glycans showed the largest mobility shift suggesting full binding towards FcγRIIa (i.e. expected binding for an IgG1 molecule). mAb-A with only one complex type N-glycan showed a lower, but still significant shift in the electrophoretic mobility indicating lower affinity to the FcγRIIa (approximately half compared to the completely glycosylated IgG molecule). For the non-glycosylated mAb-A only a very minor shift in the electrophoretic mobility was observed suggesting only very weak binding to the FcγRIIa (**Figures 2B** and **S3**).

Furthermore, the sample also contained antibodies with only the initial building blocks of the N-glycan core structure. The antibody with only two N-acetylglucosamines (GlcNAc) shows already a higher affinity to the FcγRIIa compared to the aglycosylated antibody. An addition of one mannose further increases the binding affinity. Similar binding was observed for the antibody with two GlcNAcs on each heavy chain. Yet these structures have a significantly lower affinity compared to the antibody with one complete complex-type glycan (**Figure S3**). To corroborate that the observed electrophoretic mobility shifts are related to the specific binding of specific glycoforms we also analyzed mAb-B containing three amino acid exchanges, two lysines by an alanine and one proline by a glycine (LALA-PG). In contrast to the non-glycosylated mAb, which showed very minor binding to the FcγRIIa the LALA-PG modification showed close to zero affinity of the antibody for the FcγRIIa with no mobility shift upon addition of FcγRIIa (**Figures 2C, D** **)**, demonstrating that the results in **Figures 2A, B** are representatives of binding interactions.

### Influence of glycan structure on FcγRIIa-binding affinity

Due to the high resolution provided by the MS detection, our approach permitted distinction between different glycoforms on binding. Therefore, next to the different N-glycosylation occupancy, we also investigated various IgG1 antibodies containing a range of various complex-type and mannose-type glycoforms.

**Figure 3:**
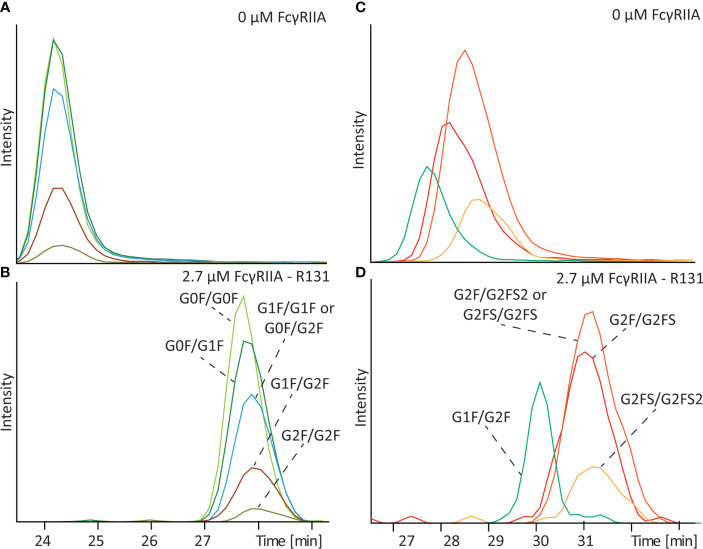
Affinity CE-MS analysis of mAb-C with **(A)** 0 µM FcγRIIa or **(B)** with 2.7 µM FcγRIIa and mAb-D (glycoengineered) mixed with mAb-D (wildtype) with **(C)** 0 µM FcγRIIa or **(D)** with 2.7 µM FcγRIIa. For clarity only G1F/G2F of the mAb-D (wildtype) is shown as an extracted ion electropherogram. For a complete overview of all glycan species in the sample please compare **Figure 5**.

To investigate the influence of galactosylation we analyzed a standard IgG1 antibody (mAb-C) containing a range of various complex-type glycoforms with different number of terminal galactoses (**Figure 3**). Analysis of the antibody in absence of receptor in the BGE resulted in co-migration of various glycoforms due to their equal electrophoretic mobility (**Figure 3A**). After addition of 2.7 µM FcγRIIa a clear shift of the electrophoretic mobility compared to the analysis without FcγRIIa was observed for all the glycoforms (**Figures 3B** and **S3**). Between glycan structures with different levels of galactose a slightly different mobility shift and hence affinity was observed. The (G0F/G0F) glycoform showed the lowest affinity, with a gradual increase in affinity with increasing number of terminal galactoses. The glycoform containing on both sides a complex-type glycan with two terminal galactoses (G2F/G2F) showed the highest affinity. Same results were observed after replication of the analysis (**Figure S4**). Analyzing mAb-B, which contains the LALA-PG mutation, with and without FcγRIIa did not show any shift or any difference between glycoforms containing different levels of galactosylation (**Figure S5**) indicating that the observed shift in mAb-C is not a methodological artefact.

To determine the influence of IgG1 Fc sialylation on affinity to FcγRIIa a glycoengineered version of mAb-D, which contained mainly sialylated antibodies (compare **Table S1**), was analyzed in combination with mAb-D (wildtype). As shown in **Figure 3C**, a separation between sialylated and non-sialylated glycoforms was observed without FcγRIIa due to their difference charge, with the neutral G1F/G2F migrating in first place followed by the antibody containing one, two or three sialic acids. After filling the capillary with 2.7 µM FcγRIIa, all glycoforms undergo a shift in their mobility indicating again binding to the FcγRIIa (**Figures 3D** and **S6**). Compared to the G1F/G2F glycoform a slightly higher mobility shift was observed for G2F/G2FS (1.5 x 10^-9^ m^2^V^-1^s^-1^ for G1F/G2F and 1.8 x 10^-9^ m^2^V^-1^s^-1^ for G2F/G2FS) suggesting some degree of influence of the sialic acid on binding. Controversially, the shift observed for G2FS/G2FS (1.6 x 10^-9^ m^2^V^-1^s^-1^) and G2FS/G2FS2 (1.4 x 10^-9^ m^2^V^-1^s^-1^) was slightly lower compared to G2F/G2FS suggesting that the addition of a second or third sialic acid may lower the binding compared to the addition of a single sialic acid.

**Figure 4:**
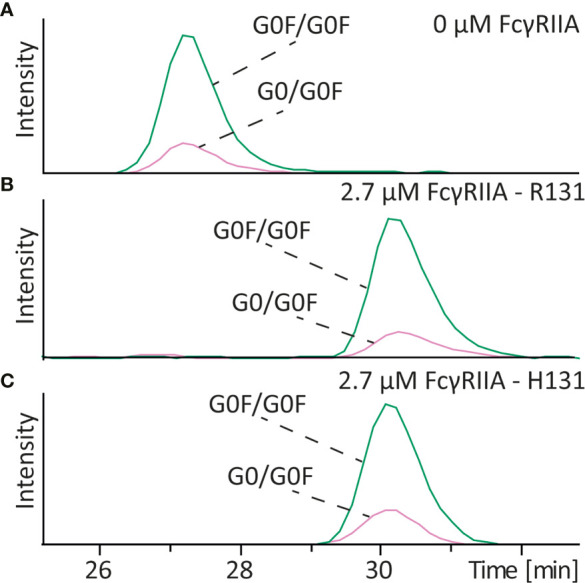
Affinity CE-MS analysis of mAb-D with **(A)** 0 µM FcγRIIa or **(B)** with 2.7 µM of the R131 variant and **(C)** 2.7 µM of the H131 variant of the FcγRIIa receptor. The figure shows extracted ion electropherograms of G0F/G0F and G0/G0F.

Core fucosylation of IgG antibodies is known to have a very strong influence on the affinity to the FcγRIII, with complex type glycans without core fucosylation having the highest affinity. To assess if this holds true for the FcγRIIa receptor we analyzed various antibodies with and without core fucosylation on different glycan structures. For mAb-D (wildtype) which contained G0F/G0F and G0/G0F structures no influence on the affinity to FcγRIIa R131 after addition of FcγRIIa was observed (**Figures 4** and **S6**). Because two different variants of the FcγRIIa (H131 and R131) exist in the human population we also studied the affinity of the different glycoforms to the H131 receptor. Same results were observed for the FcγRIIa H131 variant. Additionally, we analyzed a glycoengineered antibody mAb-E (low fucose) which was produced with 2-FF to obtain antibodies containing mainly complex type glycans without core fucosylation (compare **Table S1**). Mixing that antibody with its wildtype version also showed no difference in binding of the G0/G0 glycoform compared to the G0F/G0F glycoform to the FcγRIIa H131 (**Figures S7A, B**), neither when compared G0/G1 and G1/G1 with G0F/G1F and G1F/G1F (**Figures S8A, B** **)**. To also evaluate fucosylation in the context of high amounts of galactose a glycoengineered mAb with high amounts of galactose (mAb-E high galactose) and another with high galactose but absence of core fucose (mAb-E high galactose + low fucose) (compare **Table S1**) were mixed and analyzed in the presence and absence of FcγRIIa H131. Both samples showed binding to the FcγRIIa H131 however no difference between different levels of fucosylation on binding (**Figures S7B, C**).

**Figure 5:**
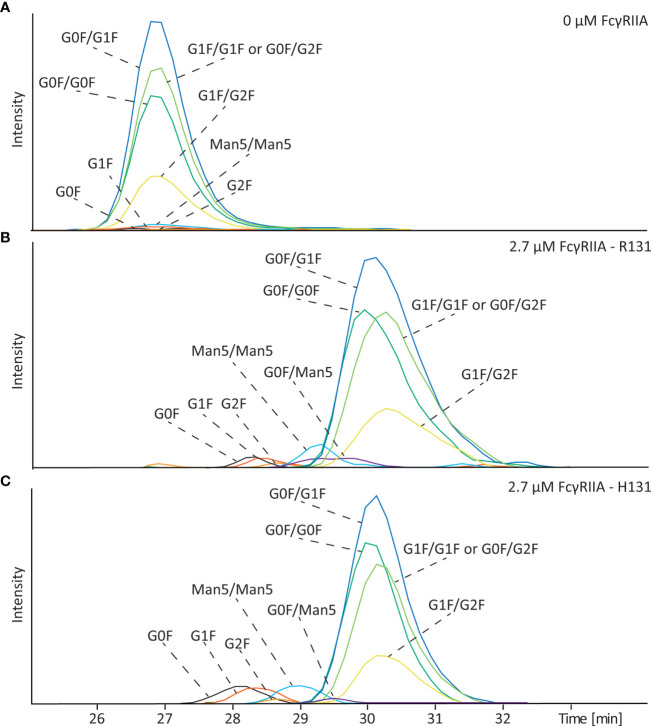
Affinity CE-MS analysis of mAb-D (wildtype) with **(A)** 0 µM FcγRIIa or **(B)** with 2.7 µM of the R131 variant and **(C)** 2.7 µM of the H131 variant of the FcγRIIa receptor. Shown are extracted ion electropherograms of the glycoforms observed in the antibody sample.

Mannose-type glycoforms are often encountered in pharmaceutical antibodies but their influence on binding to FcγRIIa has not been yet evaluated. To determine the impact of this type of glycosylation on the FcγRIIa we analyzed mAb-D (wildtype) which contained a mixture of complex-type and mannose-type glycoforms (Man5). The Man5 glycoforms are neutral and therefore have the same electrophoretic mobility than the complex-type in absence of FcγRIIa (**Figure 5A**). After analysis with 2.7 µM FcγRIIa in the BGE the antibody with only one complex type glycan showed the lowest affinity as also shown in **Figures 2** and **S3**. Here, the effect of galactosylation of complex-type glycans on binding becomes more evident with the G0F species having the lower binding.

For mannosylated species clear differences in binding were observed. The antibody with Man5/Man5 glycans on both sides shows a lower mobility shift and thereby a lower affinity than the antibody with complex type glycosylation, such as G0F/G0F (**Figures 5B** and **S6**). Interestingly, the signal of the G0F/Man5 is detected in between the signal of the G0F/G0F and the Man5/Man5 glycoform, meaning that indeed both sides of the antibody are important to obtain a strong affinity. A representative mass spectrum of the detected glycoforms of mAb-D is shown in **Figure S9**. Because mAb-D (wildtype) contained only a total of 3.1% Man5 (**Table S1**) we spiked the sample with glycoengineered mAb-D (high mannose) which contained mainly Man5/Man5 in a ratio of 1:3 (mAb-D (high mannose): mAb-D (wildtype)) to confirm that the observed effect is not influenced by the relative abundance of the species. As shown in **Figure S10**, similar decrease on the electrophoretic mobility was observed for the Man5/Man5 species in the enriched sample in comparison to the mAb-D (wildtype) containing only low amounts. Similarly, in the case of the FcγRIIa H131 variant mAb-D mannose-type species showed a clear decrease on binding affinity compared to complex-type glycoforms (**Figures 5C** and **S6** **)**. This result is especially of importance for therapeutic antibodies, which can have significant amounts of Man5.

## Discussion

Binding assessment of specific protein variants or proteoforms remains a big technical challenge. In the antibody field, developments in glycoengineering strategies have permitted to study the influence of glycosylation in binding with various FcRs in more in detail (18, 20, 23). Yet, glycoengineered antibodies are not pure glycoforms, as also shown in **Table S1** and often bring ambiguities due to the presence of other glycoforms and PTMs that can potentially influence the binding. Monitoring relative binding of individual proteoforms or glycoforms from a complex mixture provides unique opportunities to discern their differences. Affinity CE-MS strategy allows to assign the relative affinities not only of specific glycoforms, but also of truncated glycoforms, hemi-glycosylated and aglycosylated antibodies without the need of tedious production *via* glycoengineering or fractionation of different species. Individual glycoforms are resolved by MS at the composition level, allowing to assess dozens – and potentially even more – proteoforms and glycosylation variants in a single experiment independently of their relative abundance (26, 27). While structural information is obtained in the gas phase, biomolecular interactions are probed in-solution allowing affinity assessment. Furthermore, due to the high selectivity of the approach small differences in affinity (often unresolved by standard binding techniques) can be monitored. Another benefit of the approach is that minimal amounts of antibody and receptor (only few µg) are necessary to determine the affinity allowing to study the interaction of IgGs with receptors where only limited amounts of receptor are available.

In line with earlier results, we observed only very minor binding of aglycosylated IgG for FcγRIIa (14, 28), whereas the LALA-PG showed no binding to the FcγRIIa receptor (14). Interestingly, for the antibody containing only GlcNAc either on one or on both sides a reduction in binding to the FcγRIIa could be observed however stronger than compared to the non-glycosylated antibody binding. Such an effect has previously been reported for FcγRIIIa (29), however was never studied for the FcγRIIa receptor. A more recent study on IgG confirmed this finding and showed that the binding affinity for only one GlcNAc to the FcγRIIIa receptor is lower compared to an entire complex type glycan, however not completely abolished (30). They also demonstrated the importance of the GlcNAc residue to stabilize the C’E loop of the IgG molecule, which is critical for the FcγR interaction (31). Also, a report by Krapp et al. shows that partial removal of sugar moieties lead to a more and more closing of the antibody structure, which will also affect FcγR binding (32). We also found an increased affinity with only two GlcNAc per IgG molecule compared to the completely deglycosylated IgG molecule. This is the first time, that this was observed for the interaction with the FcγRIIa and furthermore we could observe an increase in affinity for 4 GlcNAc or an additional mannose, which might be due to an additional stabilization of the C’E loop and therefore enhanced accessibility for the FcγRIIa.

Similarly, we found that the hemi-glycosylated antibodies have a lower binding affinity to the FcγRIIa compared to the fully glycosylated antibody. Even though the binding of the FcγRIIa and the antibody is either in a 1:1 (33, 34) or an asymmetric 2:1 (one FcγR dimer binds one mAb) (35) ratio both glycans are necessary to obtain a high affinity. This was also shown in a report of Ha et al. where they isolated hemi-glycosylated antibodies using cation exchange chromatography and found in SPR measurements that the affinity to both FcγRIIa allotypes is decreased by half (K_D_ approximately 2 times higher) (36). Most likely this decreased binding affinity is connected to the decrease in thermal stability of hemi-glycosylated antibodies, which also might impact the structure of the CH2 domain.

Next to glycan occupancy, we could study the influence of specific glycans on binding. Certain glycan features, such as afucosylation are known to have a strong influence on the binding to the FcγRIII receptor. However, we observed no binding difference between afucosylated variants for neither FcγRIIa allotype, which is in agreement with other studies (15). Also, in the context of higher galactosylation no influence of afucosylation on the binding to the FcγRIIa could be found. Galactosylation of complex type glycans was reported to show a slight increase in binding to the FcγRIIa with the highest affinity for the antibody with two G2F glycans (20, 23, 37). In our analysis we confirmed that increase of galactosylation enhances the affinity to the FcγRIIa. For sialylation the literature is less conclusive, which might be due to the fact that sialic acids can be linked differently to the terminal galactose (2,3 vs 2,6 linkage depending of expression system) and thereby show a different binding. Whereas some reports suggest an increase in binding to the FcγRIIa (23) or a slight increase (20) other reports see either an increase or a decrease depending on the presence of core fucose and bisection (18). This example shows already the urge to look at each glycoform specifically, as glycoengineering still result in a mixture of different glycoforms. For sialylation we also observed an increase in affinity to the FcγRIIa. Interestingly, based on our data it seems that the IgG with only one sialic acid has a slightly stronger shift suggesting a higher affinity than compared to the IgGs with two or three sialic acids. A similar effect was also observed by SPR and FcgRIIa using glycoengineered IgG variants (20, 23). Contrary, using a range of 1-72% sialylation in differently engineered antibodies with regard to bisection, fucosylation and galactosylation, Dekkers et al. found no effect of sialic acid content of IgG1 on FcγRIIa binding, while a slight decrease of FcγRIIIa/b binding was found for the afucosylated and bisected variants. A minor effect with this regards was also found comparing binding of FcγRIIa-H131 to fucosylated, non-bisected and sialylated IgG1 with afucosylated, bisected and non-sialylated IgG1 (18). However the differences observed in these reports, including our study, are very minor and might be due to experimental errors. Furthermore different sialic acid linkages (2,3 versus 2,6) might have a different binding behavior. While 2,3 linked sialic acids are often present in biopharmaceuticals sialic acids are 2,6 linked in naturally occurring human antibodies and their behavior will be addressed in future studies. Overall can be noted that all glycoengineered samples are never pure species showing the great advantage of our assay to determine the affinity of each specific glycoform in a mixture without the need of any prefractionation or glycoengineering.

High mannose glycoforms showed a decrease in affinity, which was so far never reported for the FcγRIIa. The effect was the strongest when two high mannose glycans were combined on the Fc portion and less, but still lower compared to G0F/G0F when one Man5 structure was in combined with a complex type glycan. This decrease in affinity might be a result of a decrease in the antibody stability (38). This might also affect the structure of the CH2 domain and therefore explain the decrease in affinity. Most likely is the structure of the Man5/G0F glycan not so dramatically influenced as the one with Man5/Man5. This finding is of utmost importance especially of therapeutic IgG1 samples, which can contain higher amounts of Man5 structures. Similarly to our finding, Man5 glycoforms were recently shown to have a reduced binding to FcγRIIIa compared to the afucosylated complex type glycans (39). Therefore the expansion of our platform to other receptors such as FcγRIIIa is warranted to support this finding.

## Data availability statement

The original contributions presented in the study are included in the article/Supplementary materials, further inquiries can be directed to the corresponding author/s.

## Author contributions

CG performed the measurements and processed the data. ED-V conceived the idea and designed the experiments. AK and TS provided the Fc receptors, DR and GV provided the mAb samples. CG and ED-V drafted the manuscript. CG, GV, AK, TS, DR, MW, and ED-V reviewed this manuscript equally.

## Funding

This work was supported by the Analytics for Biologics project (Grant agreement ID 765502) of the European Commission and the LUMC Fellowship 2020 to ED-V.

## Conflict of interest

Authors DR, TS, AK are employed by the company Roche Diagnostics GmbH, Penzberg, Germany.

The remaining authors declare that the research was conducted in the absence of any commercial or financial relationships that could be construed as a potential conflict of interest.

## Publisher’s note

All claims expressed in this article are solely those of the authors and do not necessarily represent those of their affiliated organizations, or those of the publisher, the editors and the reviewers. Any product that may be evaluated in this article, or claim that may be made by its manufacturer, is not guaranteed or endorsed by the publisher.
