# Supplementary material for: Affinity capillary electrophoresis – mass spectrometry permits direct binding assessment of IgG and FcγRIIa in a glycoform-resolved manner
Source: Front Immunol. 2022 Sep 8;13:980291. doi: 10.3389/fimmu.2022.980291 (PMC9494200; doi:10.3389/fimmu.2022.980291)
Supplement: Supplementary file 1 [file DataSheet_1.docx]

Supplementary Material

Affinity capillary electrophoresis – mass spectrometry permits direct binding assessment of IgG and FcγRIIa in a glycoform-resolved manner

**Christoph Gstöttner^1^, Alexander Knaupp^2^, Gestur Vidarsson^3^, Dietmar Reusch^4^, Tilman Schlothauer^2^, Manfred Wuhrer^1^, Elena Domínguez-Vega^1^***

^1^Leiden University Medical Center, Center for Proteomics and Metabolomics, Leiden, The Netherlands.

^2^Pharma Research and Early Development, Roche Innovation Center Munich, Germany.

^3^Department of Experimental Immunohematology, Sanquin Research and Landsteiner Laboratory,

Amsterdam UMC, University of Amsterdam, Amsterdam, The Netherlands

^4^Pharma Technical Development Penzberg, Roche Diagnostics GmbH, Penzberg, Germany.

*** Correspondence:**Elena Domínguez Vega
e.dominguez_vega@lumc.nl

## Supplementary Figures


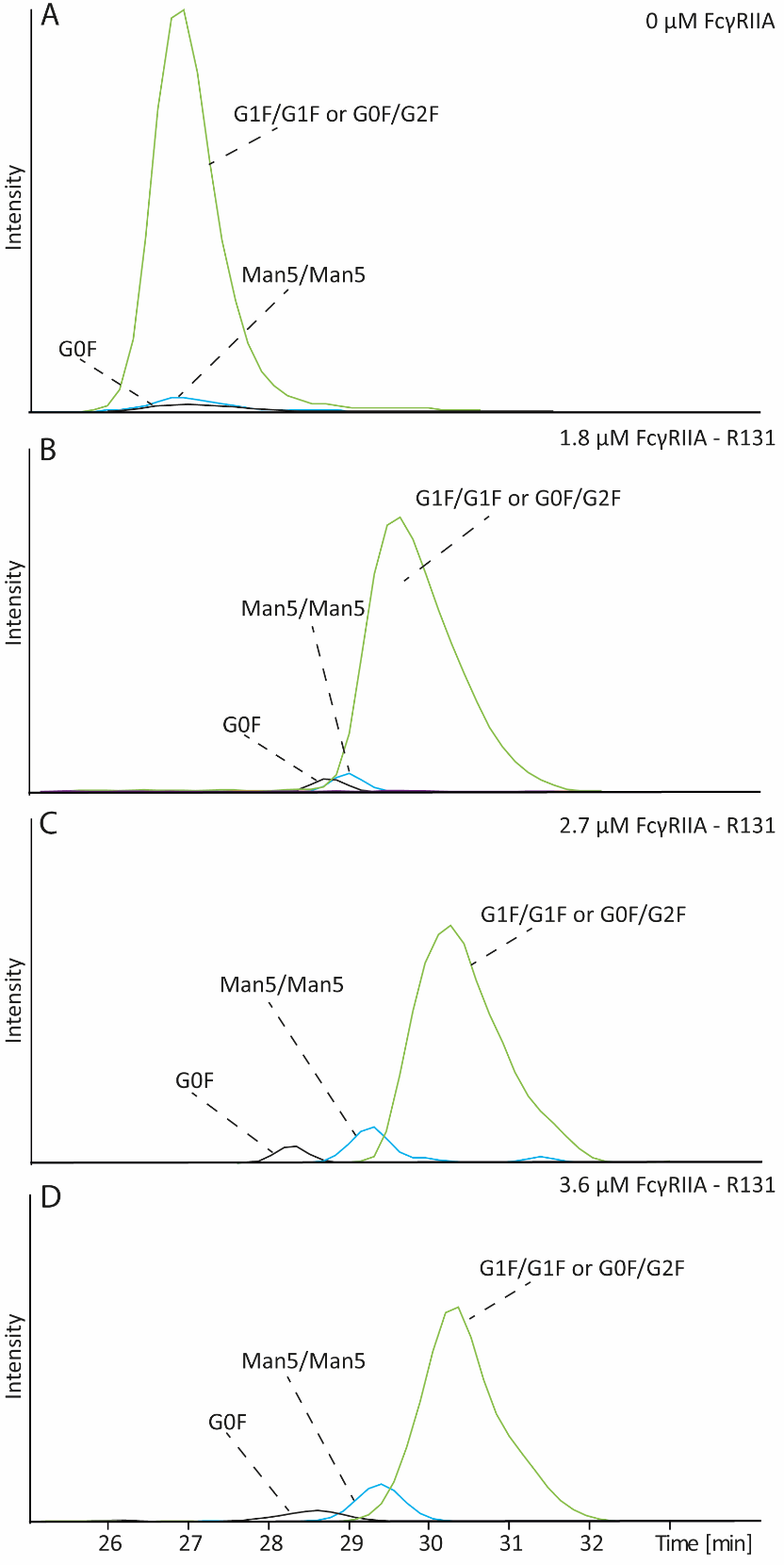


**Figure S1**: Affinity CE-MS analysis of mAb-D with A) 0 µM FcγRIIa, B) with 1.8 µM FcγRIIa, C) 2.7 µM FcγRIIa and D) 3.6 µM FcγRIIa. Different colors represent EIEs of mAb-D with different glycoforms.


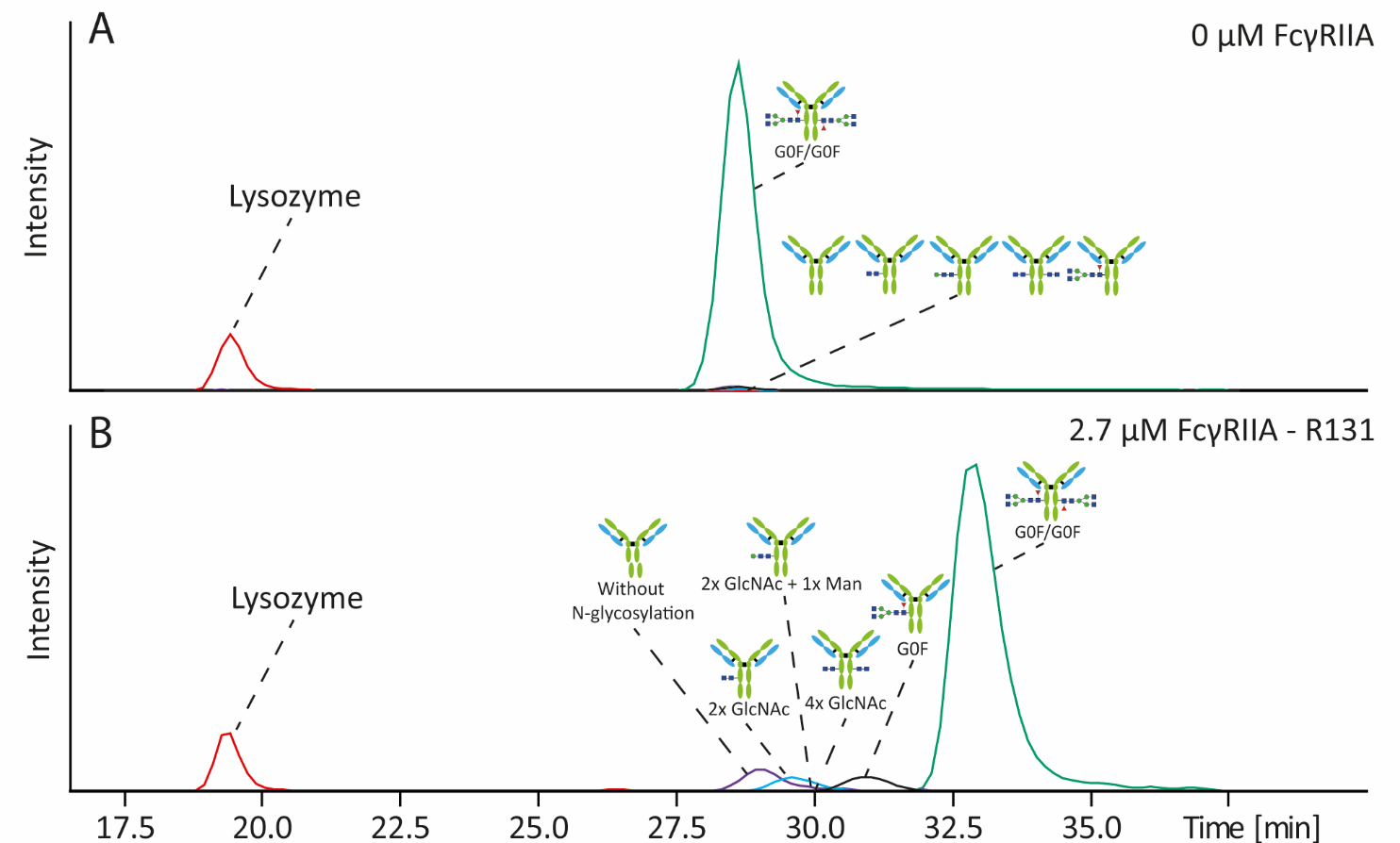


**Figure S2**: Affinity CE-MS analysis of mAb-A with A) 0 µM FcγRIIa or B) with 2.7 µM FcγRIIa. Marker protein Lysozyme (red line) used to align the between analysis. Different colors represent EIEs of the illustrated antibody molecules with different degree of glycosylation.


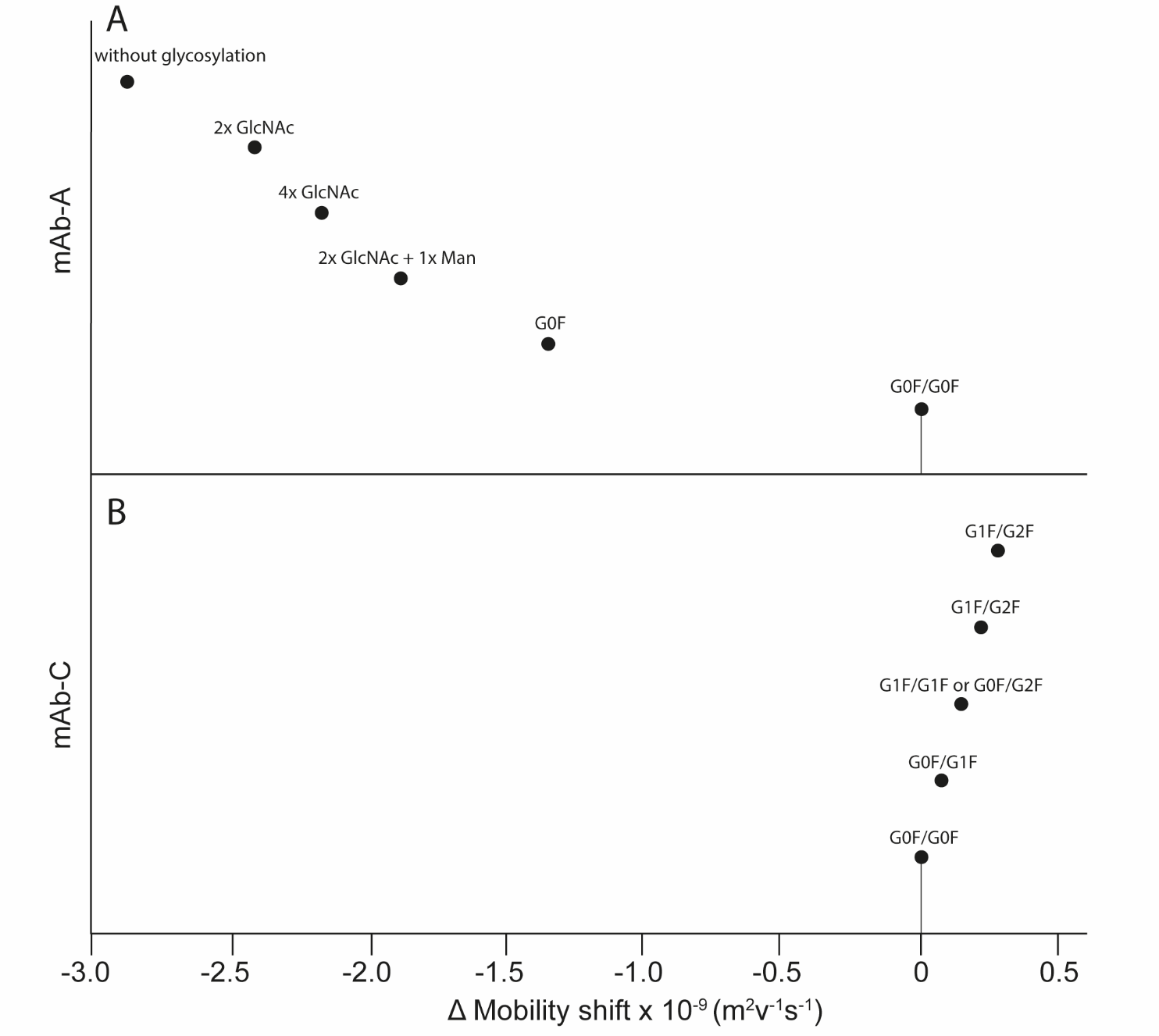


**Figure S3**: Differences in mobility shift of A) mAb-A and B) mAb-C. Differences in mobility shifts were calculated using G0F/G0F as a reference.


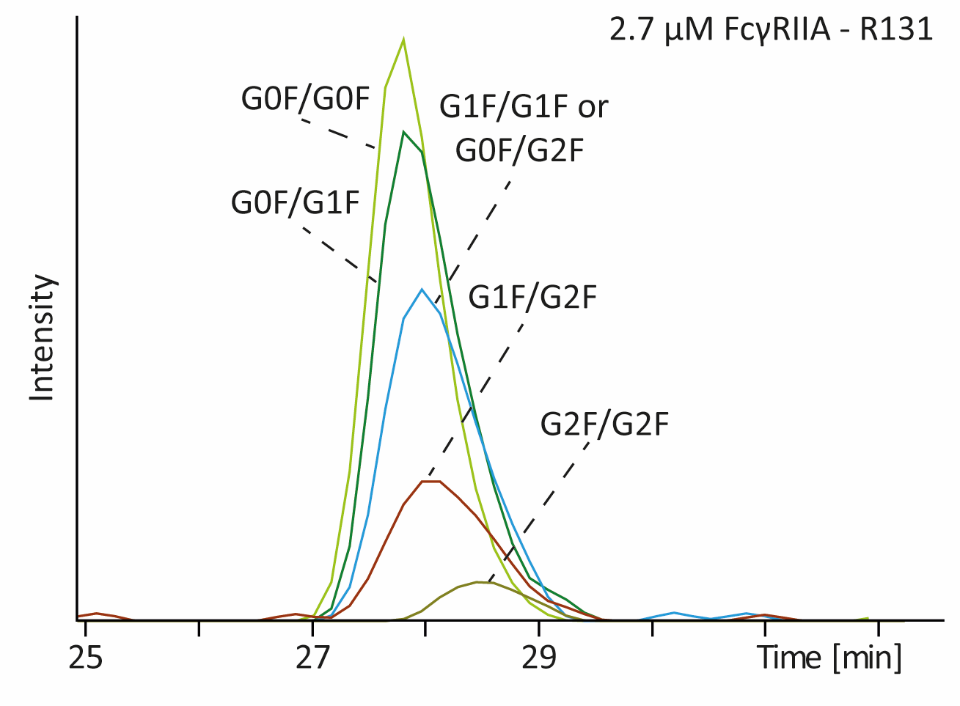


**Figure S4**: Replicate affinity CE-MS analysis of mAb-C with A) 0 µM FcγRIIa or B) with 2.7 µM FcγRIIa. Different colors represent EIEs of mAb-C with different degree of galactosylation.


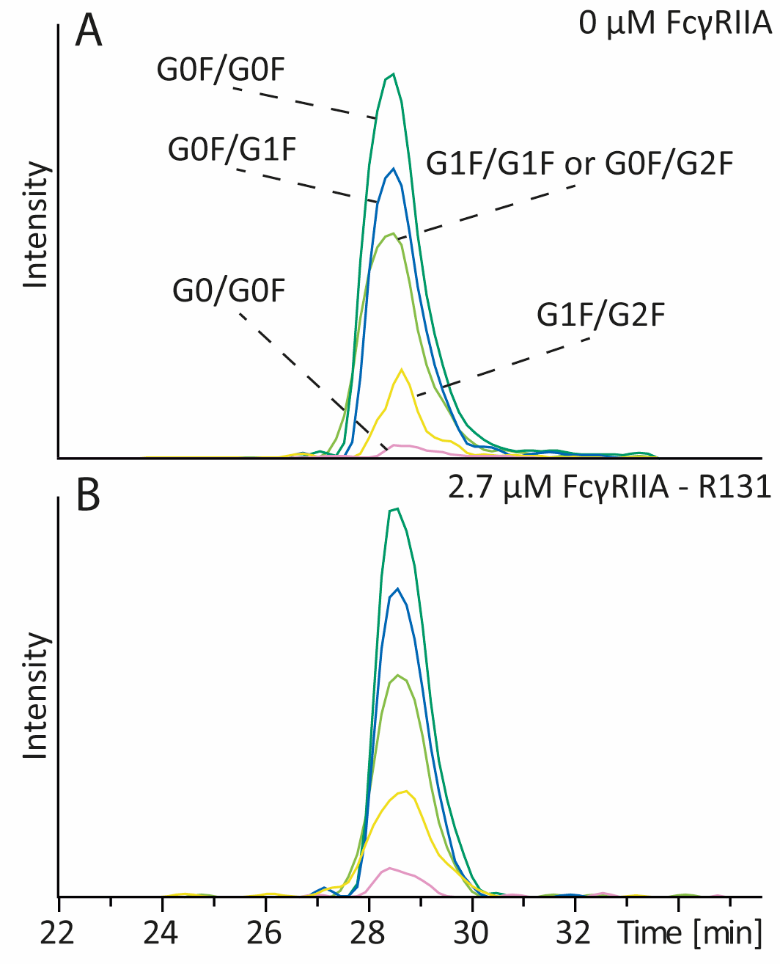


**Figure S5**: Affinity CE-MS analysis of mAb-B containing a LALA-PG mutation with A) 0 µM FcγRIIa or B) with 2.7 µM FcγRIIa. Different colors represent EIEs of mAb-B with different degree of galactosylation.


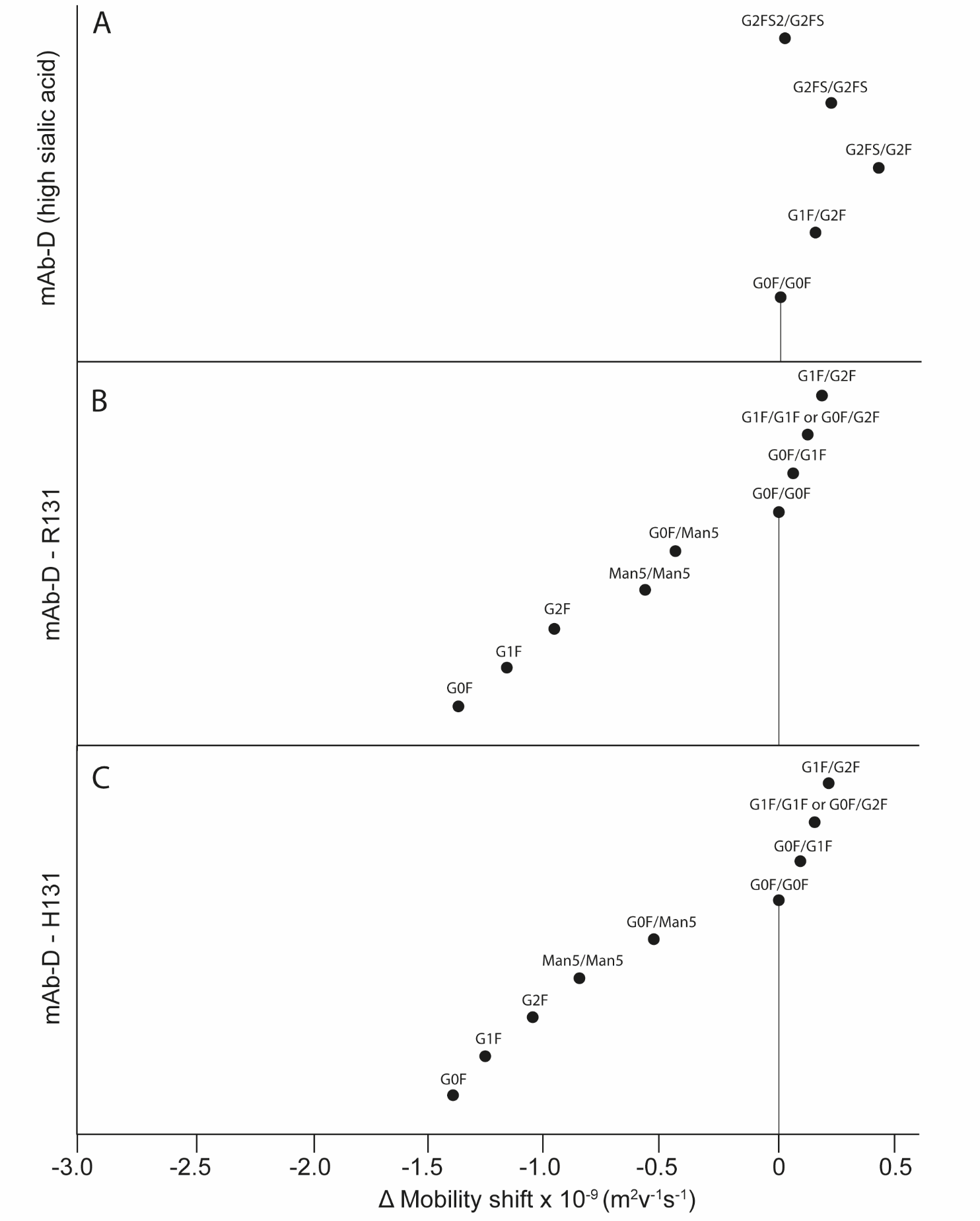


**Figure S6**: Differences in mobility shift of A) mAb-D (high sialic acid), B) mAb-D (wildtype) analyzed with FcγRIIa R131 and C) mAb-D (wildtype analyzed with FcγRIIa H131. Differences in mobility shifts were calculated using G0F/G0F as a reference.


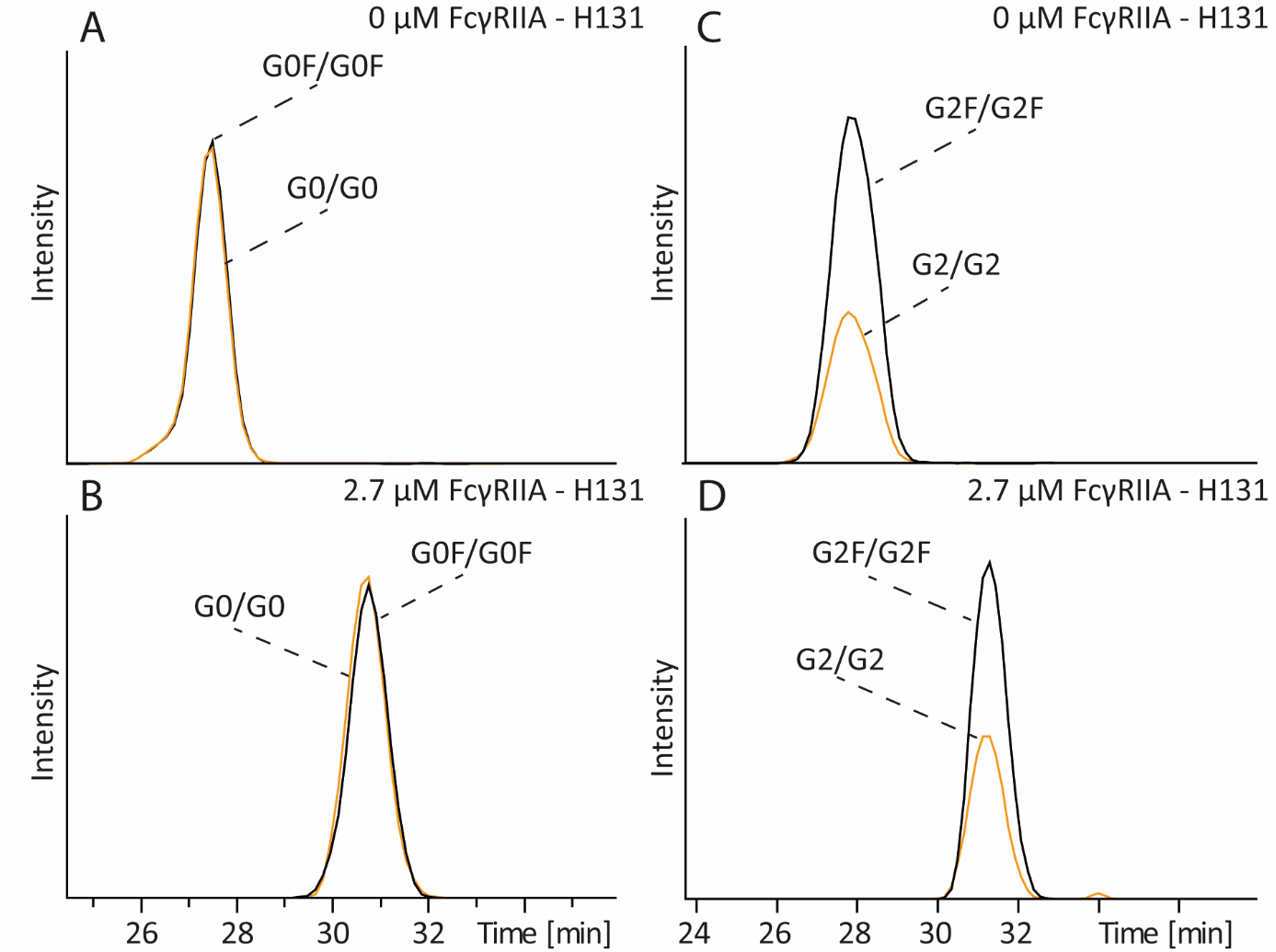


**Figure S7**: Affinity CE-MS analysis of A) mAb-E (wildtype) mixed with mAb-E (low fucose) with 0 µM FcγRIIa or B) with 2.7 µM FcγRIIa. C) shows the analysis of a mixture of mAb-E (high galactose) and mAb-E (high galactose + low fucose) with 0 µM FcγRIIa or D) with 2.7 µM FcγRIIa. Shown are extracted ion electropherograms of the main glycoforms.


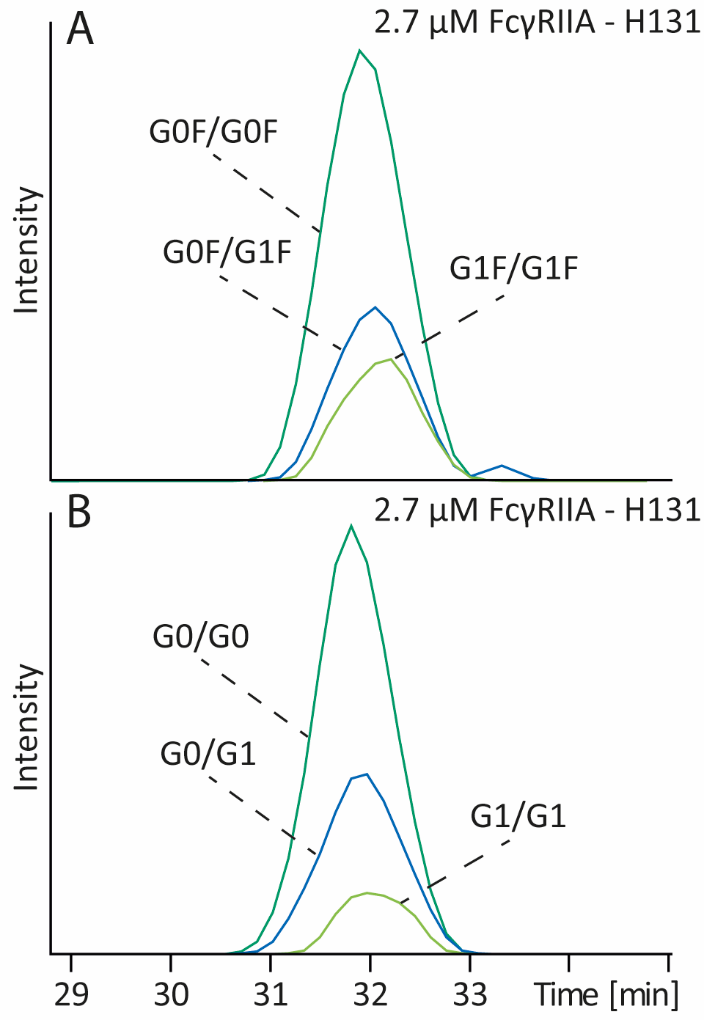


**Figure S8**: Affinity CE-MS analysis with 2.7 µM FcγRIIa of A) mAb-E (wildtype) or mAb-E (low fucose). Shown are extracted ion electropherograms of the main glycoforms.


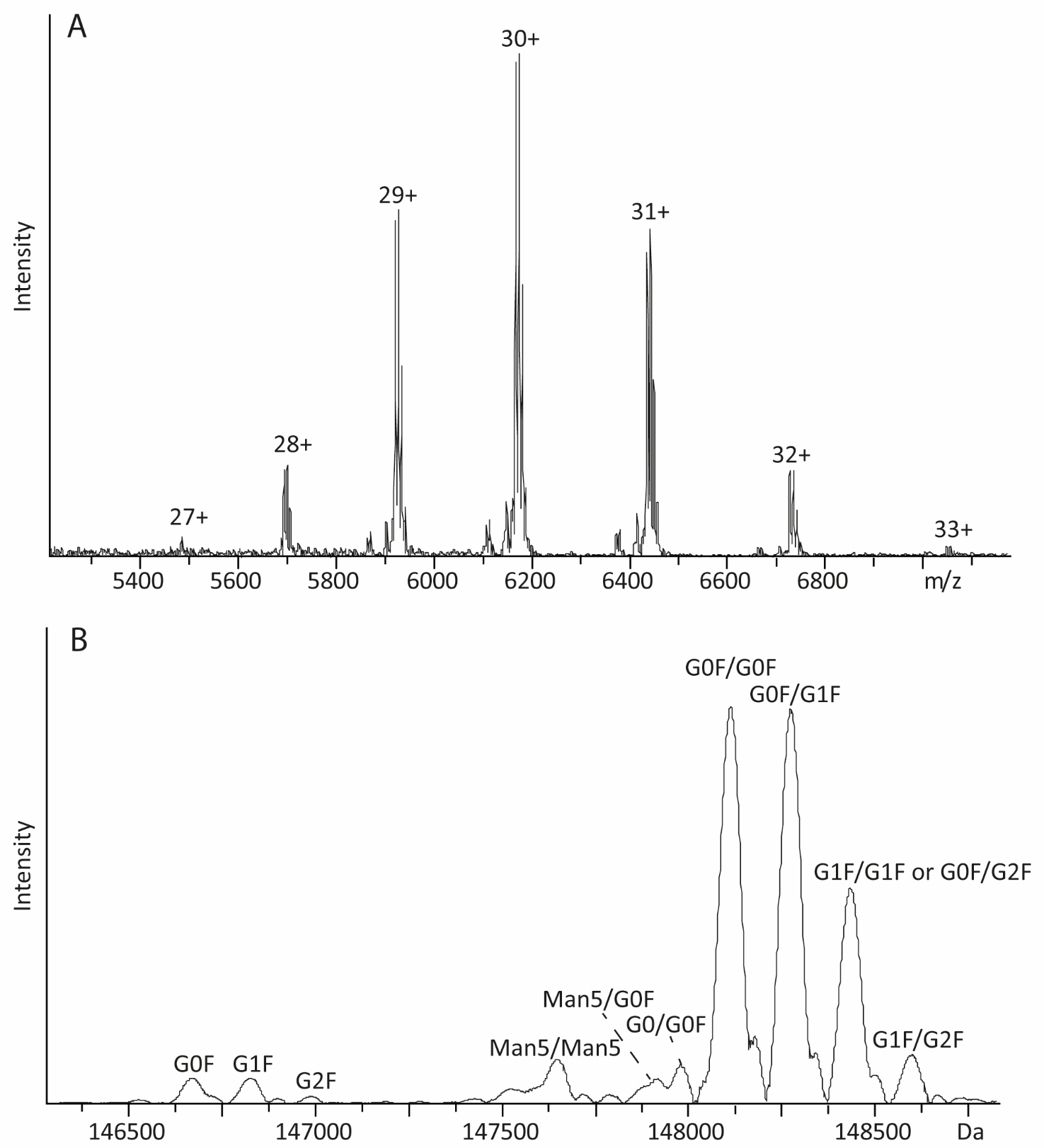


**Figure S9**: Representative mass spectrum for the analysis of mAb-D. A) charge state distribution of mAb-D and B) deconvulted mass spectrum with the assignment of the different glycoforms.

­
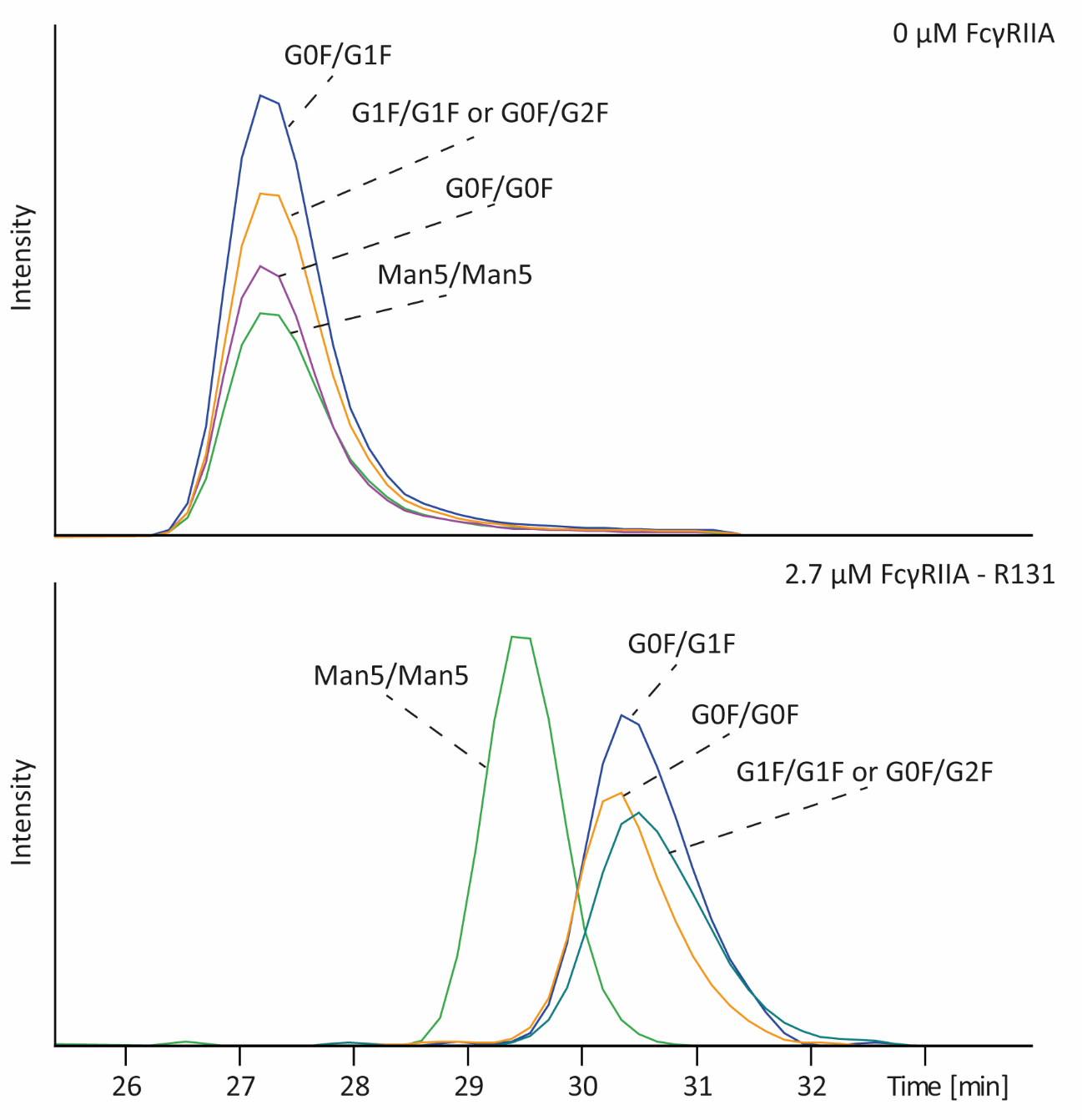


**Figure S10**: Affinity CE-MS analysis of mAb-D (wildtype) spiked with mAb-D (high mannose) in a ratio of 1:3 (mAb-D (high mannose) : mAb-D (wildtype)) with A) 0 µM FcγRIIa or B) with 2.7 µM FcγRIIa. Different colors represent EIEs of different mAb-D glycoforms.

**Table S1**: Glycopeptide data for mAb-D (wildtype) [1], (high sialic acids) [2] and (high mannose) [3], as well as mAb-E (wildtype), (low fucose), (high galactose) and (high galactose and high fucose) [4] analyzed by electrospray (ESI) MS. n.d.: not detected.

| Glycoform | Relative Abundance (%) | | | | | | |
| --- | --- | --- | --- | --- | --- | --- | --- |
|  | mAb-D  (wildtype) | mAb-D  (high sialic acid) | mAb-D  (high mannose) | mAb-E  (wildtype) | mAb-E  (low fucose) | mAb-E  (high galactose) | mAb-E  (high galactose + low fucose) |
| H5N4F1 | 9.3 | 1.3 | 0.7 | 7.2 | 1.0 | 60.7 | 9.5 |
| H6N3F1 | n.d. | n.d. | n.d | 0.2 | n.d. | 2.1 | 0.2 |
| H8N2 | n.d. | n.d. | n.d | 0.1 | 0.1 | 0.1 | 0.2 |
| H3N5F1 | n.d. | n.d. | n.d | 1.3 | 0.2 | 0.1 | n.d. |
| H5N4 | 0.6% | n.d. | 0.2 | 0.1 | 3.1 | 0.2 | 58.4 |
| H4N4F1 | 39.2 | 0.6 | 4.2 | 28.1 | 4.6 | 7.8 | 1.0 |
| H6N3 | n.d. | n.d. | 0.3 | 0.1 | 0.2 | 1.4 | 3.4 |
| H5N3F1 | n.d. | n.d. | 0.1 | 0.7 | 0.1 | 1.1 | 0.2 |
| H7N2 | n.d. | n.d. | n.d | 0.1 | 0.2 | 0.1 | 0.2 |
| H4N4 | 4.8 | n.d. | 0.7 | 0.1 | 19.1 | n.d. | 7.8 |
| H3N4F1 | 32.4 | n.d. | 5.4 | 53.8 | 7.8 | 4.2 | 0.4 |
| H5N3 | n.d. | n.d. | 0.5 | 0.4 | 0.9 | 0.2 | 1.4 |
| H4N3F1 | 0.9 | n.d. | 0.4 | 0.4 | 0.1 | 0.7 | 0.1 |
| H6N2 | 0.2 | n.d. | 0.3 | 0.1 | 0.1 | 0.1 | 0.1 |
| H3N4 | 6.6 | n.d. | 1.1 | 0.3 | 57.1 | n.d. | 4.2 |
| H4N3 | n.d. | n.d. | 0.2 | 0.1 | 0.6 | n.d. | 0.9 |
| H3N3F1 | 1.2 | n.d. | 1.1 | 1.3 | 0.2 | 0.2 | n.d. |
| H5N2 | 3.1 | 1.2 | 81.6 | 2.7 | 2.5 | 2.4 | 2.3 |
| H4N2 | n.d. | n.d. | 2.0 | n.d. | n.d. | n.d. | n.d. |
| H3N2 | n.d. | n.d. | 0.2 | n.d. | n.d. | n.d. | n.d. |
| H3N3 | 0.9 | n.d. | 0.4 | 0.1 | 1.2 | n.d. | 0.1 |
| H5N4F1S2 | < 0.1 | 27.0 | n.d | 0.1 | n.d. | 2.2 | 0.5 |
| H5N4F1S1 | 0.4 | 60.4 | 0.1 | 0.7 | 0.2 | 10.8 | 1.6 |
| H5N5F1 | n.d. | n.d. | n.d | 0.2 | 0.1 | 0.3 | n.d. |
| H4N4F1S1 | 0.1 | n.d. | < 0.1 | 0.3 | 0.1 | 0.1 | n.d. |
| H5N3F1S1 | n.d. | n.d. | 0.1 | 0.1 | n.d. | 4.9 | 0.6 |
| H4N5F1 | n.d. | n.d. | n.d | 1.1 | 0.2 | 0.3 | n.d. |
| H5N4S1 | n.d. | n.d. | n.d | n.d | 0.3 | n.d. | 6.6 |
| H4N4S1 | n.d. | n.d. | n.d | n.d. | 0.2 | 0.1 | 0.1 |
| H4N3F1S1 | n.d. | 3.2 | 0.3 | n.d. | n.d. | n.d. | n.d. |
| H4N3S1 | n.d. | 0.9 | 0.2 | n.d. | n.d. | n.d. | n.d. |
| H5N4S1 | n.d. | 4.0 | n.d | n.d. | n.d. | n.d. | n.d. |
| H5N4S2 | n.d. | 1.6 | n.d | n.d. | n.d. | n.d. | n.d. |

**References**

[1] D. Reusch, M. Haberger, D. Falck, B. Peter, B. Maier, J. Gassner, M. Hook, K. Wagner, L. Bonnington, P. Bulau, M. Wuhrer, Comparison of methods for the analysis of therapeutic immunoglobulin G Fc-glycosylation profiles-Part 2: Mass spectrometric methods, MAbs 7(4) (2015) 732-42.

[2] N. de Haan, K.R. Reiding, M. Haberger, D. Reusch, D. Falck, M. Wuhrer, Linkage-Specific Sialic Acid Derivatization for MALDI-TOF-MS Profiling of IgG Glycopeptides, Analytical Chemistry 87(16) (2015) 8284-8291.

[3] D. Falck, M. Thomann, M. Lechmann, C.A.M. Koeleman, S. Malik, C. Jany, M. Wuhrer, D. Reusch, Glycoform-resolved pharmacokinetic studies in a rat model employing glycoengineered variants of a therapeutic monoclonal antibody, MAbs 13(1) (2021) 1865596.

[4] W. Hoepel, H.-J. Chen, C.E. Geyer, S. Allahverdiyeva, X.D. Manz, S.W. de Taeye, J. Aman, L. Mes, M. Steenhuis, G.R. Griffith, P.I. Bonta, P.J.M. Brouwer, T.G. Caniels, K. van der Straten, K. Golebski, R.E. Jonkers, M.D. Larsen, F. Linty, J. Nouta, C.P.A.A. van Roomen, F.E.H.P. van Baarle, C.M. van Drunen, G. Wolbink, A.P.J. Vlaar, G.J. de Bree, R.W. Sanders, L. Willemsen, A.E. Neele, D. van de Beek, T. Rispens, M. Wuhrer, H.J. Bogaard, M.J. van Gils, G. Vidarsson, M. de Winther, J. den Dunnen, High titers and low fucosylation of early human anti–SARS-CoV-2 IgG promote inflammation by alveolar macrophages, Science Translational Medicine 13(596) (2021) eabf8654.
